# A method for exploring implicit concept relatedness in biomedical knowledge network

**DOI:** 10.1186/s12859-016-1131-5

**Published:** 2016-07-19

**Authors:** Tian Bai, Leiguang Gong, Ye Wang, Yan Wang, Casimir A. Kulikowski, Lan Huang

**Affiliations:** 1 College of Computer Science and Technology, Jilin Univesity, 2699 Qianjin St, Changchun, China; 2 Key Laboratory of Symbolic Computation and Knowledge Engineering of Ministry of Education, Jilin University, 2699 Qianjin St, Changchun, China; 3 Yantai Intelligent Information Technologies Ltd., 2699 Qianjin St, Yantai, China; 4 Department of Computer Science, Rutgers, The State University of New Jersey, 2699 Qianjin St, Piscataway, NJ USA

**Keywords:** Biomedical ontology, Knowledge network, Implicit relatedness

## Abstract

**Background:**

Biomedical information and knowledge, structural and non-structural, stored in different repositories can be semantically connected to form a hybrid knowledge network. How to compute relatedness between concepts and discover valuable but implicit information or knowledge from it effectively and efficiently is of paramount importance for precision medicine, and a major challenge facing the biomedical research community.

**Results:**

In this study, a hybrid biomedical knowledge network is constructed by linking concepts across multiple biomedical ontologies as well as non-structural biomedical knowledge sources. To discover implicit relatedness between concepts in ontologies for which potentially valuable relationships (implicit knowledge) may exist, we developed a Multi-Ontology Relatedness Model (MORM) within the knowledge network, for which a relatedness network (RN) is defined and computed across multiple ontologies using a formal inference mechanism of set-theoretic operations. Semantic constraints are designed and implemented to prune the search space of the relatedness network.

**Conclusions:**

Experiments to test examples of several biomedical applications have been carried out, and the evaluation of the results showed an encouraging potential of the proposed approach to biomedical knowledge discovery.

## Background

Precision medicine [1] has become a most promising methodology for clinical medicine, which relies heavily on rich biomedical knowledge and information of individual patients such as genetic content, living habits, environmental factors, etc. [2]. US National Academy of Sciences claims in a 2011 research report that a biomedical knowledge network based on biological data and knowledge is necessary for precision medicine [3]. How to compute relatedness between concepts and discover valuable information and implicit knowledge effectively and efficiently from such hybrid knowledge (both structural and non-structural) networks is a key of paramount importance to the realization of precision medicine, and a huge challenge facing the biomedical research community. It is agreeable that the knowledge network should include all the knowledge sources, information systems and repositories in biomedicine available today and in the future, spanning the whole spectrum of structural and non-structural information and knowledge.

One type of important knowledge sources is ontology. Knowledge represented in biomedical ontology systems such as Gene Ontology [4] and Disease Ontology [5] is a conceptualization of agreed-upon observations or findings in a domain of the actual world, and is structural to logically represent the taxonomical relationships between biomedical entities (concepts) as well as other semantic (e.g. causal) relationships of various kinds. Such knowledge in general can be considered as explicit knowledge. However, when a biomedical ontology system becomes very large, or multiple ontologies are being studied together, the semantic relatedness of two biomedical entities not directly connected may not be easy to see or understand. For example, the relatedness between a gene and a disease may not be easily or directly observable as a simple causal relationship. Yet, this type of implicit relatedness can be “interesting” and potentially valuable for it may lead us to the discovery and the establishment of important relationship between the two. Different from explicit knowledge, implicit knowledge in general, though there appears to be no generally agreed-upon definition for it [6–8], may be characterized in terms of weak relationships between biomedical entities, for example, a disease may be often mistaken as another disease (misdiagnosis), or a disease may share similar symptoms with another, etc. Relationships of this nature may often seem to be of small significance, but some of them may reveal or imply important, though implicit, relatedness between the biomedical entities, providing valuable information or knowledge in revealing deeper and more subtle connections between the related biomedical entities.

A great number of research studies in working with biomedical knowledge or information have been focusing on mining knowledge from massive genomics data. Very limited efforts have been devoted to exploring implicit knowledge using biomedical ontologies. All these works, though have made important progress in biomedical knowledge discovery, do not provide a unified and systematic approach to the problem of implicit knowledge discovery within the knowledge networks of multiple and different biomedical knowledge sources, thus limiting their ability to combine both structural and non-structural knowledge and information sources in discovering hidden or implicit relatedness between biomedical entities.

To overcome or at least alleviate the above mentioned shortcoming, we define a novel unified computational framework based on a network of biomedical entities across multiple and different knowledge and information sources such as disease ontology, gene ontology and PubMed, linked using inter-relationships between them. Based on this model, we propose a new measurement of concept relatedness, and develop a set-theoretic inference scheme to compute a network of relatedness between concepts that may uncover valuable and implicit relationships (implicit knowledge) between them. We provide a systematic evaluation of the method using experimental results of example applications in biomedicine.

The main contribution of our work is the formulation and implementation of a unified computational framework based on which a hybrid biomedical knowledge network can be structural, concept relatedness and relatedness networks can be computed using a formal inference mechanism based on set-theoretic operations.

## Related work

Most existing biomedical knowledge repositories can be classified into two categories: non-structural (e.g. research papers) and structural (e.g. semantic network, knowledge graph, ontologies, etc.). Research on knowledge representation and discovery with these two types of knowledge has been making encouraging progresses in recent years.

**Non-structural biomedical knowledge discovery** Literature is a main form of non-structural knowledge, such as research publications, clinical guidelines, clinical trials, and reports of case studies. Increasing efforts have been made to extract various types of disease-related knowledge from these relatively unstructural materials. Liu and Hu [9] developed a distant supervised model to extract gene expression relationship between genes and brain regions from literature. Marwah et al. [10] implemented a context-specific Bayesian framework for computing functional relationships as links between ontologies, based on the statistics of co-occurrence of terms in the literature. Xu et al.’s work [11] focuses on extracting disease-manifestation relationships from the literature, while De la Iglesia et al. [12] deal with ontology concept extraction in the context of classification of clinical trial information. According to Seyfang et al. [13] and Isern et al. [14], ontologies can be developed to represent formal guidelines. Cheng et al. [15] have also made progress in establishing semantic associations among disease related databases to provide a more global view of human diseases.

**Semantic network and semantic web** Semantic Network [16] is a network representing knowledge in terms of concepts and their semantic relations. WordNet [17] is one of well-known examples of semantic network. Non-Axiomatic Reasoning System (NARS) [18] also represents knowledge in the form of network. Semantic Web [19], on the other hand, provides a common framework over the Web for knowledge sharing and reuse across applications, enterprises, and community boundaries. Chen et al. [20] conduct fruitful research on semantic web based biomedical data analysis.

**Knowledge graph** Knowledge Graph (KG) is a representational model proposed by Google to capture and graphically represent the semantics of real-world entities and their relationships [21], which supports more informative keyword based search. A number of knowledge graphs have been built, such as YAGO [22], DBpedia [23], NELL [24], Freebase [25]. Efforts have been made to build biomedical knowledge bases in the form of KG [26].

**Artificial Neural Network** Artificial Neural Network and Deep Learning have made a significant leap in the performance of AI systems. For example, the 152-layer neural network developed by Microsoft Research Asia achieves an error rate of 3.57 % on the test set of ImageNet [27]. Recently, AlphaGo, a computer Go program that uses ‘value networks’ to evaluate board positions and ‘policy networks’ to select moves, defeated the human world Go champion [28]. However, the cover story of a recently published Science Magazine pointed out that, people learning new concepts can often generalize successfully from just a single example based on already learned knowledge, yet machine learning algorithms typically require tens or hundreds of examples to perform with similar accuracy [29].

**Biomedical knowledge representation and discovery in ontology** Ontology is a main form of structural knowledge system, and a formal, explicit specification of shared conceptualization [30]. It’s main function is sharing and reuse of knowledge [31]. Many biomedical ontology systems have been built such as Gene Ontology [4], Disease Ontology [5], Human Phenotype Ontology [32], Environment Ontology [33], Protein Ontology [34], etc. Mohammed et al. [35] align the Diseases Ontology with the Symptoms Ontology by exploring links between diseases and symptoms. Concepts in these and other biomedical ontologies are organized primarily using hierarchical “is-a” relationships, while other valuable relationships such as “may-have-complication” and “may-have-side-effect” are mostly missing for they are usually weak and statistical in nature. For knowledge reuse, techniques like ontology mapping [36] and ontology alignment [37] enable us to bridge different biomedical ontologies by identifying concepts that share the same meaning. Some research studies ontology systems for a specific domain by applying network structure analysis. Wang et al. [38] propose a Network Ontology Analysis (NOA) method to perform gene ontology enrichment analysis on biological networks. Weng and Chang [39] apply the technique of ontology network analysis to document recommendations. Other studies, like Chen [8] and Liu et al. [40], analyze ontology networks by applying methods developed for complex networks or social network.

The above mentioned research and many other similar studies in structural knowledge representation and discovery are mostly focusing on the development of new biomedical knowledge systems and improvement of the existing ones. These systems to date remain independent or even isolated from one another. Furthermore, most existing works with multiple ontologies are exploring direct and explicit relationships between concepts by mapping and integrating different ontologies. Much less attention has been paid to the development of a unified knowledge representation framework linking semantically all biomedical knowledge ontologies. In recent years, work on integrating different knowledge repositories (both structural and non-structural) to explore indirect relatedness between concepts starts emerging. Corinna Vehlow et al. [41] developed a method to visualize and analysis of existing knowledge (from databases and the literature) and experimental data together in a network model. Spangler and Han et al. [42, 43] focus on mining relevance between heterogeneous biomedical entities from literature. These studies mostly use statistical methods (e.g. co-occurrence) to explore relationships between concepts. However, important relationships between concepts sometimes can only be revealed by examining indirect relatedness.

In this paper, a network based biomedical knowledge representation framework and a corresponding computational model are proposed to address the issue of implicit relatedness computing.

## Methods

We developed a unified computational model based on a hybrid biomedical knowledge network of linked biomedical concepts across multiple different knowledge and information sources. It consists of biomedical ontologies as well as other biomedical information and knowledge repositories such as PubMed. This system was constructed using relationships between concepts from each respective knowledge source (e.g. disease ontology, gene ontology, and online biomedical publication repositories). To discover interesting relatedness between concepts for which potential valuable relationships (implicit knowledge) may exist, a new measurement of relatedness and a new set-theoretic inference scheme were also proposed.

### Construction of biomedical knowledge network (BMKN)

**Fig. 1:**
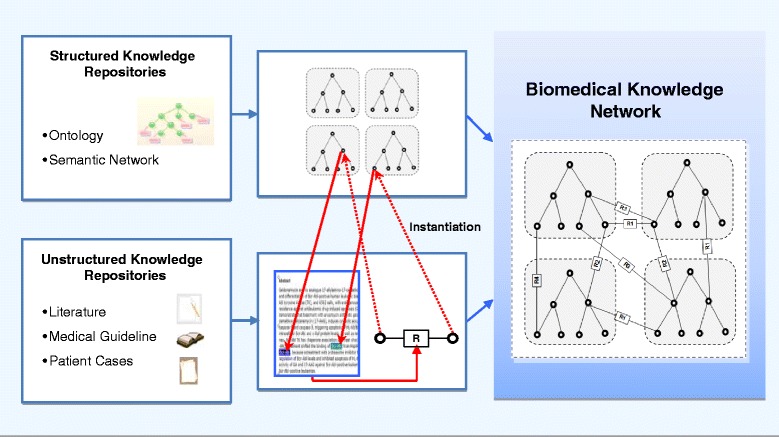
Biomedical knowledge network construction

Biomedical knowledge represented and managed in different knowledge repositories in general can be classified as structural knowledge such as ontology, semantic web and knowledge graph, and non-structural knowledge such as research papers, medical case reports, and text books. Relationships between biomedical concepts can be searched and extracted from these repositories. Many important concepts in the structural repositories like ontology have direct mapping in the non-structural repositories like literature. For instance, concept of “breast cancer” in disease ontology is also mentioned and discussed in many research papers. A specific concept like “breast cancer” in a research paper can be viewed as an instance of the same concept in the disease ontology. We can also further generalize this mapping by treating the instance of concept “breast cancer” and the context (e.g. the paper) within which it is discussed as an instantiation of the concept in disease ontology. Using this type of mapping or instance-of relationship, we linked ontologies to non-structural repositories. Using relationships between concepts across multiple knowledge sources, we constructed the system containing linked biomedical ontologies and non-structural knowledge repositories. As shown in Fig. 1, relationships between concepts within an ontology system are extracted from structural knowledge repositories, and relationships between ontologies are extracted from non-structural knowledge repositories. Concepts appeared in both an ontology system and a paper in a non-structural repository are mapped through an instance-of relationship.

A Biomedical Knowldge Network (BMKN) can be formally defined as a directed graph $G = \left ({V,E,C,S} \right)$, where *V* denotes a node of concept, *E* denotes a link of relationship between concepts, *C* denotes a confidence factor of the link and *S* denotes a significance factor of the link. A concept-ontology mapping is defined as *Φ*:*V*→*O* and function *Φ*(*v*)∈*O* gives a specific ontology for a specific concept *v*. Relationship mapping function is defined as *Ψ*:*E*→*R*, and *Ψ*(*e*)∈*R* gives a specific relationship for link *e*(*u,v*)∈*E*. Relationships can be extracted from both non-structural (e.g. research publications) and structural knowledge sources (ontologies). A confidence factor for a link *e* is defined as $C\left (e \right)$, representing the probability that link or edge *e* of relationship may exist between concept *u* and concept *v*. For edges extracted from non-structural repositories, the confidence values are computed by normalized frequency of co-occurence (TF-IDF). For links extracted from structural knowledge sources, the existing or given confidence values are used. For ontologies, a default confidence value 1 for edges is chosen. Significance value of an link $S\left (e \right)$ computed from non-structural knowledge is defined, in our experiments with the framework, in terms of the impact factors of the publication where concepts *u* and *v* co-occurenced. Similarly, the significance factors computed from structured knowledge sources are given a default value. Our framework also support other measurement of the significance factor in terms of the importance of relationship.

Once the knowledge network is constructed, exploration of implicit relatedness between concepts within and across multiple ontologies can be carried out based on a computational model we developed called MORM.

### Multi ontology relatedness model (MORM)

There are different types of semantic relationships in the constructed BMKN, e.g. has-symptom, regulate, etc. These relationships between biomedical concepts or entities are expressions of known biological processes within the human body. How to use these explicit relationships to further explore and uncover indirect or implicit relationships is still an open issue. For instance, if a specific disease entity DO1 in disease ontology has a relationship with an entity GO1 in a gene ontology system due to the biomedical function of GO1, then it would be very meaningful and interesting to question if some similar/related diseases of DO1 may have an implicit relationship with similar/related concepts of GO1. However, such type of implicit relationships has not been formulated at the level of abstraction such that they can be treated systematically in exploring indirect or implicit relationships between biological concepts. In MORM model, we generalize and treat all the different types of relationships as one of “relatedness of concepts”.

An intra-relationship *R*_intra_ is defined as a generic relationship of any kind between two concepts within a same ontology.

An inter-relationship *R*_inter_ is defined as a generic relationship of any kind between two concepts across multiple ontologies.

They are defined as follow: ∀*e*(*u,v*)∈*E*, *Φ*(*u*)=*O*_*i*_, *Φ*(*v*)=*O*_*j*_

$$\left\{ \begin{array}{ll} \Psi (e) = R_{\text{intra}}, &if~i = \mathrm{j}\\ \Psi (e) = R_{\text{inter}}, &if~i \ne j \end{array} \right.$$

In MORM, multiple related ontologies are represented as a connected network of concepts via both intra-relationships within a same biomedical ontology (e.g. lung cancer is-a respiratory system cancer in a disease ontology), and inter-relationships across multiple biomedical ontologies (e.g. lung cancer has-symptom-of cough across both a disease ontology and a symptom ontology). In this model, semantic relatedness between one concept and all of its semantically connected concepts is represented in terms of a Relatedness Network (RN), and computed using a general inference mechanism based on a set-theoretic method.

A RN is a graph of linked concepts. A link in this graph is either of the intra-relationship or inter-relationship type. The relatedness between any two concepts are measured and computed by MORM’s inference mechanism.

**Fig. 2:**
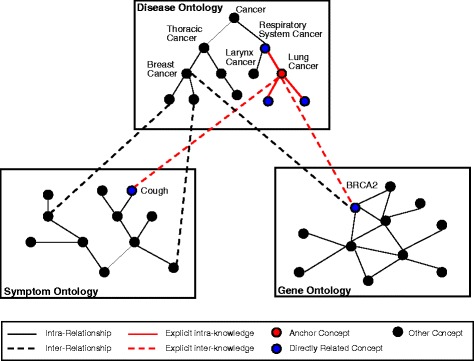
Biomedical ontologies in MORM

As an example shown in Fig. 2, a RN is computed by linking the concepts in three different biomedical ontologies through both intra-relationship (*R*_intra_) and inter-relationship (*R*_inter_) relationships. A concept node (in red) in the disease ontology, called an “anchor concept”, directly connects (red link) to other three concepts (in blue) within the disease ontology (intra-relationship) and to two concepts (in blue) in the symptom ontology and gene ontology respectively (inter-relationship). These directly linked concepts and their relationships together form a semantic structure we can easily see and interpret, representing a type of explicit knowledge. In this example, the node of lung cancer is directly linked to the node of respiratory system cancer (*R*_intra_) and we can easily see and interpret it as lung cancer being a type of respiratory system cancer. Similarly, the connection of *R*_inter_ type between the node of lung cancer and a blue node of cough in the symptom ontology, and the connection of *R*_inter_ type between lung cancer and a blue node of BRCA2 in the gene ontology represent a type of explicit knowledge that can also be easily observed and interpreted.

Any two concepts may also be connected indirectly via multiple links and nodes (biomedical entities) representing indirect or implicit relationships of both the *R*_intra_ and *R*_inter_ types. As shown in Fig. 2, the node of lung cancer is indirectly connected to the larynx cancer node via the node of respiratory system cancer, and also connected indirectly to breast cancer via the node of gene BRCA2. Such relationships may not be so easy or obvious to interpret what can be inferred or learned. The point to make is that the relatedness of these cancers may provide valuable hints for discovering new information or knowledge about them. Such knowledge in the present biomedical ontologies can only be called implicit knowledge.

Implicit knowledge discovery by associating explicit knowledge is a common practice in medical research. According to Swanson [44], dietary fish oil is found to lead to certain blood and vascular changes, and other related research reported abnormally high blood viscosity been found in patients with Raynaud’s disease. Associating these two findings together implies that fish oil might benefit Raynaud’s disease patients. Years later, results of a clinical trial supported this earlier hypothesis [45].

Relatednesses between biomedical concepts that proved to be useful and interesting from a research perspective are usually domain-specific. MORM computationally defines concept relatedness in terms of the RN network, within which the interestingness of relatedness between the anchor concept and any other concept can be further evaluated in the context of a specific domain. One advantage of MORM model is that it provides a general platform and inference mechanism based on which other domain specific inference strategies or constrains can be designed and applied as we will discuss in a later section.

### Computing the relatedness network

In this section, a formal inference mechanism based on set-theoretic operations is proposed to compute the relatedness network. For the sake of clarity and without losing its generality, we will describe the method in details using a two-ontology network first, and then generalize it for multi-ontology network.

In the two-ontology situation, a MORM model contains Disease ontology and Symptom ontology as an example, expressed as *D* and *S* respectively. Let ontology *D* contains *n* concepts, expressed as *D*={*D*_1_,*D*_2_,…,*D*_*n*_}, while ontology *S* contains *m* concepts, expressed as *S*={*S*_1_,*S*_2_,…,*S*_*m*_}.

The following notations are used:

- *D*_*x*_ and *D*_*y*_ are two concepts in ontology *D*, the link of intra-relationship(R_*intra*_) is expressed as *D*(*D*_*x*_→*D*_*y*_).
- *S*_*x*_ and *S*_*y*_ are two concepts in ontology *S*, the link of intra-relationship (R_*intra*_) is expressed as *S*(*S*_*x*_→*S*_*y*_).
- The link of inter-relationship (R_*inter*_) of *D*_*x*_ from ontology *D* and *S*_*y*_ from ontology *S* is expressed as *H*(*D*_*x*_→*S*_*y*_).

*D*(*D*_*x*_→*D*_*y*_), *S*(*S*_*x*_→*S*_*y*_), *H*(*D*_*x*_→*S*_*y*_) are links of relatedness indicating explicit knowledge.

We introduce a set theoretic method: General Inference Mechanism (GIM).

First, we define a set operators as R_*intra*_ and R_*inter*_ for the set operations within ontology *D* or *S*, and across ontologies *D* and *S*, respectively.

In ontology *D*, *D*^′^ is defined as a set of concepts in ontology *D*. *D*(*D*_*x*_→*D*_*y*_), denotes the link of intra-relationship (R_*intra*_) of *D*_*x*_ and *D*_*y*_. Then, *D*^′^·R_*intra*_ means the set of concepts in ontology *D* that have explicit intra-relationship with *D*^′^, which can be expressed as

1 $$ D' \cdot \mathrm{R}_{intra} = \{ {D_{y}}\left| D({D_{x}} \to {D_{y}}) \right.,{D_{x}} \in D',{D_{y}} \in D\}  $$

Similarly, we can define the set operation within ontology *S*, and that across ontologies *D* and *S*, respectively, as follows:

2 $$ S' \cdot \mathrm{R}_{intra} = \{ {S_{y}}\left| {S({S_{x}} \to {S_{y}})} \right.,{S_{x}} \in S',{S_{y}} \in S\}  $$

3 $$ D' \cdot \mathrm{R}_{inter} = \{ {S_{y}}\left| {H({D_{x}} \to {S_{y}})} \right.,{D_{x}} \in D',{S_{y}} \in S\}  $$

We then generalize the formula for relatedness network:

4 $$ {~}^{i}{D^{j}} = \left\{ \begin{array}{ll} {~}^{i - 1}{D^{j}} \cup {~}^{i}{D^{j - 1}}&i = j\\ \\ {~}^{i}{D^{j - 1}} \cdot \mathrm{R}_{intra} \cup {~}^{i}{D^{j - 1}}&i < j\\ \\ {~}^{i - 1}{S^{i - 1}} \cdot \mathrm{R}_{inter} \cup {~}^{i - 1}{D^{j}} &i > j \end{array} \right.  $$

where ^*i*^*S*^*j*^ can be computed in the same way.

**Fig. 3:**
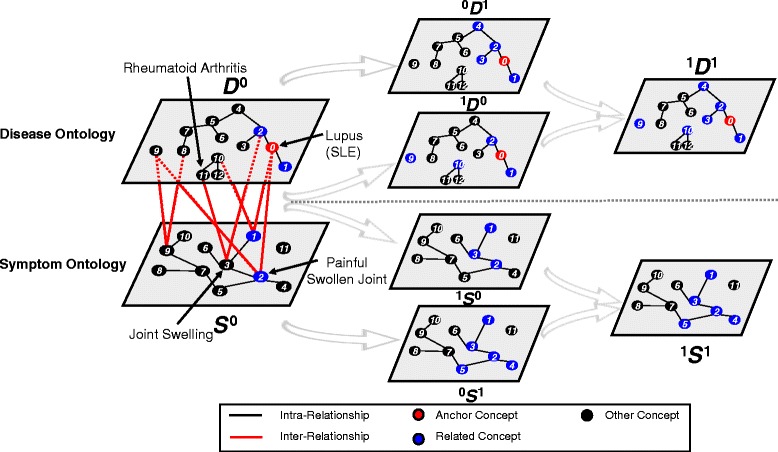
Illustration of discovering interesting relatednesses

^*i*^*D*^*j*^ is the set of concepts (nodes) collected after *j* step expansion through intra-relationship from the anchor concept, denoted as *D*_*anchor*_ (illustrated as the red node in Fig. 3), and all the concepts collected after the *i* step expansion through inter-relationship from *D*_*anchor*_. Please note, a single ontology is just a special case in our model. In this case, *i*=0, and operation for *i*<*j* applies.

Figure 3 illustrates the expansion of relatedness network step by step. Intuitively, every recursion step can be seen as the expansion of the related concept set. An initial related concept set *D*^0^ and *S*^0^ is defined as follow:

Intuitively *D*^0^ is the set of concepts that have explicit intra-relationship (R_*intra*_) with *D*_*anchor*_, indicated by the blue node 1 and 2 in ontology *D* in Fig. 3, ${D^{0}} = {D_{anchor}} \cdot \mathrm {R}_{intra} = \{ {D_{1}},{D_{2}}\} $.

Similarly *S*^0^ is defined as the set of concepts that have explicit inter-relationship (R_*inter*_) with *D*_*anchor*_, indicated by the blue nodes 1 and 2 in ontology *S* in Fig. 3, ${S^{0}} = {D_{anchor}} \cdot \mathrm {R}_{inter} = \{ {S_{1}},{S_{2}}\} $.

Concepts in sets *D*^0^ and *S*^0^ are directly connected to (via both intra- and inter-relationship) *D*_*anchor*_, presenting no interesting relatedness between them. We take the recursion (expansion of RN) one step further collecting concepts through the intra- or inter-relationship with concepts in sets *D*^0^ and *S*^0^. Since they are not directly related to *D*_*anchor*_, thus present somewhat interesting relatedness between them. We now carry the expansion process in steps as follows:

- ^0^*D*^1^ is the set of concepts that have explicit intra-relationship (R_*intra*_) with *D*^0^, plus *D*^0^ itself, as shown in Fig. 3. ${~}^{0}{D^{1}} = \{ {D^{0}} \cdot \mathrm {R}_{intra} \cup {D^{0}}\} = \{ {D_{1}},{D_{2}},{D_{3}},{D_{4}}\} $.
- ^1^*D*^0^ is the set of concepts that have explicit inter-relationship (R_*inter*_) with *S*^0^, plus *D*^0^ itself, as shown in Fig. 3. ${~}^{1}{D^{0}} = \{ {S^{0}} \cdot \mathrm {R}_{inter} \cup {D^{0}}\} = \{ {D_{1}},{D_{2}},{D_{9}},{D_{10}}\} $.
- ^1^*D*^1^ is the set of concepts that have both explicit intra- and inter-relationship (R_*intra*_ and R_*inter*_) with *D*^0^, plus *D*^0^ itself, as shown in Fig. 3. ${~}^{1}{D^{1}} = \{ {~}^{0}{D^{1}} \cup {~}^{1}{D^{0}}\} = \{ {D_{1}},{D_{2}},{D_{3}},{D_{4}},{D_{9}},{D_{10}}\} $.

^1^*D*^1^ can also be denoted as *D*^1^, indicating the first interesting relatedness step of *D*_*anchor*_.

Expansion of related concepts for ontology *S* is computed in a similar fashion:

$${~}^{0}{S^{1}} = \{ {S^{0}} \cdot \mathrm{R}_{intra} \cup {S^{0}}\} = \{ {S_{1}},{S_{2}},{S_{3}},{S_{4}},{S_{5}}\} $$

$${~}^{1}{S^{0}} = \{ {D^{0}} \cdot \mathrm{R}_{inter} \cup {S^{0}}\} = \{ {S_{1}},{S_{2}},{S_{3}}\} $$

$${~}^{1}{S^{1}} = \{ {~}^{0}{S^{1}} \cup {~}^{1}{S^{0}}\} = \{ {S_{1}},{S_{2}},{S_{3}},{S_{4}},{S_{5}}\} $$

Based on formula (4), the set of concepts in step 2 can be recursively computed as follows:

$${~}^{1}{D^{2}} = \{ {~}^{1}{D^{1}} \cdot \mathrm{R}_{intra} \cup {~}^{1}{D^{1}}\} $$

$${~}^{2}{D^{1}} = \{ {~}^{1}{S^{1}} \cdot \mathrm{R}_{inter} \cup {~}^{1}{D^{1}}\} $$

$${~}^{2}{D^{2}} = \{ {~}^{1}{D^{2}} \cup {~}^{2}{D^{1}}\} $$

We see that, ^2^*D*^2^ can be recursively computed until the recursion reaches *D*^0^ and *S*^0^, when the whole process ends.

Figure 3 actually shows an example to illustrate the inference process for search and expansion of relatedness concepts for disease ontology and symptom ontology. Lupus was cited as one of the top 10 misdiagnosed diseases [46]. As shown in Fig. 3, Lupus (Node *D*_0_ in red) has a symptom of painful swollen joint (Node *S*_2_), while Rheumatoid Arthritis (Node *D*_11_ in black) has a symptom of joint swelling (Node *S*_3_). Painful swollen joint and joint swelling are similar symptoms, and can be easily incorrectly expressed by patients. We can inferred that Rheumatoid Arthritis may be related to Lupus through the inference process of Lupus →Painful Swollen Joint →Joint Swelling →Rheumatoid Arthritis.

For multi ontologies *O*_*t*_∈{*O*_1_,*O*_2_,⋯*O*_*n*_,}, we generalize the above formalization of computing relatedness network from two ontologies {*D,S*} as follows:

5 $$ {~}^{i}{O_{t}}^{j} = \left\{ \begin{array}{ll} {~}^{i - 1}{O_{t}}^{j} \cup^{i}{O_{t}}^{j - 1} &i = j\\ {^{i}}{O_{t}}^{j - 1} \cdot \mathrm{R}_{intra} \cup {~}^{i}{O_{t}}^{j - 1} &i < j\\ \bigcup\limits_{k = 1,k \ne t}^{n} ({~}^{i - 1}{O_{k}}^{i - 1} \cdot \mathrm{R}_{inter}) \cup {~}^{i - 1}{O_{t}}^{j}&i > j \end{array} \right.  $$

${~}^{i}{O_{t}^{j}}$ is the set of concept nodes in ontology *O*_*t*_ which containing all the concepts collected after the *j* step expansion through intra-relationship from the anchor concept, and all the concepts collected after the *i* step expansion through inter-relationship from anchor concept.

### Pruning relatedness network

A relatedness network generated using the MORM model enables researchers to explore, within a controllable search space for potentially interesting implicit relatedness between an anchor concept and a collection of related concepts. Some concepts in the collection are not easily found to be related to the anchor concept using a single ontology or knowledge repository. However the space of an RN could still be too large to manage if the scale of the ontology is very large. In the MORM model, the inference mechanism also supports the inclusion of various generic and domain specific pruning strategies to constrain the scale of the RN. The pruning strategies can be applied to the ontology networks as a part of the inference process, or to a generated RN separately. In this section we will introduce two such pruning strategies or rules.

#### Computing RN using inter-relationships only

For a linked ontology network *O*_*t*_∈{*O*_1_,*O*_2_,⋯*O*_*n*_,}, let *O*_*anchor*_ denote anchor concept in *O*_*s*_. We define a pruning rule for pruning concepts collected using inter-relationships: $F(i) = {O_{t}^{i}} - {~}^{0}{O_{t}^{i}}$ where ${O_{t}^{i}}$ is the set of all the concepts collected after the *i* step inter- and intra-relatedness expansion in relation to anchor concept *O*_*anchor*_; ${~}^{0}{O_{t}^{i}}$ is the subset containing concepts collected after the *i* step intra-relatedness expansion in *O*_*t*_ with in relation to the anchor concept *O*_*anchor*_ in *O*_*s*_. Thus, by utilizing the entire ontology network instead of the single ontology alone, the search can reach the concepts that may contain implicit relatedness with *O*_*anchor*_ by excluding ${~}^{0}{O_{t}^{i}}$ from ${O_{t}^{i}}$.

When we explore concept relatedness in a network of single ontology, *O*_*anchor*_ is from *O*_*t*_, i.e. *O*_*t*_=*O*_*s*_, formula $F(i) = {O_{s}^{i}} - {~}^{0}{O_{s}^{i}}$ represents all the concepts collected after the *i* step expansion in relation to *O*_*anchor*_ excluding concepts that develop through only intra-relationship within set *O*_*s*_.

We can then denote the inference strategy in two-ontology (Disease ontology & Symptom ontology) situation as: *F*(*i*)=*D*^*i*^− ^0^*D*^*i*^

As shown in the Fig. 3, when *i*=2, *F*(2)= ^2^*D*^2^− ^0^*D*^2^={*D*_9_,*D*_10_,*D*_11_,*D*_12_}

By using this rule, the search found and collected *D*_9_,*D*_10_,*D*_11_,*D*_12_, which are not linked to *D*_*anchor*_ in set *D* or very far away from *D*_*anchor*_, being a set of concepts of interesting relatedness.

#### Pruning RN by link masking

In MORM, a RN can be computed using domain independent inference rules, which does not distinguish different types of relationships or links between concepts. To reduce the RN space, a relationship or link mask can be implemented to remove part of the network following unwanted relationships or links in the RN. If the mask is applied as a part of inference to compute RN, the inference process will then focus only on selected types of semantic relationships while ignoring masked relationships, thus potentially improving search efficiency and accuracy. More formally speaking, the masking operation is to reject a subset of relationships of type *R*^′^, from set of semantic relationships of type *R*, i.e. *Ψ*(*e*)∉*R*^′^. The inference or expansion process can mask a link based on the values of three properties of the link: relationship type, significance and confidence as we mentioned earlier. For each relationship type, various masks can be designed based on these properties. In the experiments with MORM model, we implemented a set of masks in the form of a triplet *R*(*t,c,s*) using different settings. For instance, *R*1(1,0.25,0.5) is a pruning mask for relationship type *R*1, which means for each link of type *R*1, if the confidence value is larger than 0.25 and significance value is larger than 0.5, then the link will be followed by the RN expansion process. Also, mask *R*2(0,0,0) will block the expansion process for all links of type *R*2. For this mask, if the type value is 0, the choice of values for either confidence factor or significance factor does not really matter. Other different forms of masks also can be implemented within our framework depending on the need of specific applications and users’ preference.

## Results and Discussion

In this section, we give an evaluation of our method with three experiments of example applications.

### Experiment 1: discovering interesting relatedness of possible misdiagnosis

In this experiment, we test the ability of our method to help discover possible disease misdiagnosis. The result is evaluated by comparing the found likely misdiagnoses with “differential diagnoses” listed on medical book [47].

#### Experimental design

**Step 1 Building MORM model** For this experiment, a MORM model is built containing a disease ontology DO and a symptom ontology SYMP [48], as well as the inference engine for computing the relatedness network. All concepts are organized using “is-a” relationship representing their intra-relationship, *R*_intra_. The model also contains has-symptom relationships across both ontology systems representing the inter-relationship, *R*_inter_. The inter-relationship between disease ontology and symptom ontology is extracted from medical guideline. The model includes 77,531 relatedness links for diseases, 48,841 relatedness links for symptoms, and 223,804 relatedness links between diseases and symptoms.

**Step 2 Computing “relatedness network” of possible misdiagnosis** A relatedness network containing possible misdiagnosis for a specific disease is computed by using the associated inference rules of set operation.

**Step 3 Comparative evaluation** We use the “differential diagnosis” list in book “Current Essentials of Medicine” [47] as a reference. We check how many “differential diagnosis” diseases listed in the reference can be found in step 2, denoted as *N*1. We also compute similar diseases for a specific disease by using DO alone, which means only intra-relationship within DO is used in comparison. Then, we check how many “differential diagnosis” diseases listed in the reference can be found in resulted similar diseases, denoted as *N*2. Then, we compare *N*1 and *N*2. The test repeats for 50 common diseases.

#### Result analysis

We show the comparison for 6 diseases and give average performance for all the 50 diseases in Table 1. Our method found the majority of the differential diagnosis diseases in the reference (*N*1), which is more effective than the approach using DO only (*N*2).

**Table 1:** Result of discovering misdiagnosis

	Differential diagnosis	Possible misdiagnosis diseases		Similar diseases in DO	
	diseases (number)	(*N*_1_)	(percentage)	(*N*_2_)	(percentage)
Type 1 diabetes	6	5	83.3 %	3	50.0 %
Acute pancreatitis	6	4	66.7 %	1	16.7 %
Sinusitis	5	4	80.0 %	2	40.0 %
Tuberculous meningitis	5	5	100.0 %	2	40.0 %
Cystitis	8	4	50.0 %	3	37.5 %
Acute tracheobronchitis	5	5	100.0 %	2	40.0 %
**Average for 50 common diseases**	**6.2**	**4.5**	**72.6 %**	**2.2**	**35.5 %**

Taking Type 1 Diabetes as an example, the number of differential diagnosis of it amounts to 6 according to the reference (as shown in column 1). 5 out of 6 are found by our method (or 83.3 %, as shown in column 2 and 3). As comparison, 3 out of 6 (50 %, as shown in column 4 and 5) are found by comparison approach in step 4. Thus, our approach is proved to be effective. The obvious better performance of our method is also shown for the other 5 diseases and for the 50 diseases in Table 1.

We should note that, the Relatedness Network found by our approach may contain uninteresting relatednesses also, but it also means that there may exist other meaningful relatednesses besides the misdiagnosis relatedness. For instance, there are 6 differential diagnosis diseases of type 1 diabetes in the reference, but our experiment yields more diseases that have one or more same symptoms. Diseases in our result but not in any references that are potentially meaningfully related to Type 1 Diabetes may have potential to reveal valuable information for clinical medicine.

### Experiment 2: discovering interesting relatednesses for genetic diseases

The number of diseases reported that can be related to various genes or their products is growing very rapidly with the development of new genomics technologies. Diseases related to the same or related genes or gene products may share common causal factors at the molecular level. However, these related diseases may not be classified as similar diseases in the same disease class under the current disease classification system ICD-11. In this experiment, we test our method in exposing such potential molecular-level-relationships between diseases.

#### Experimental design

**Fig. 4:**
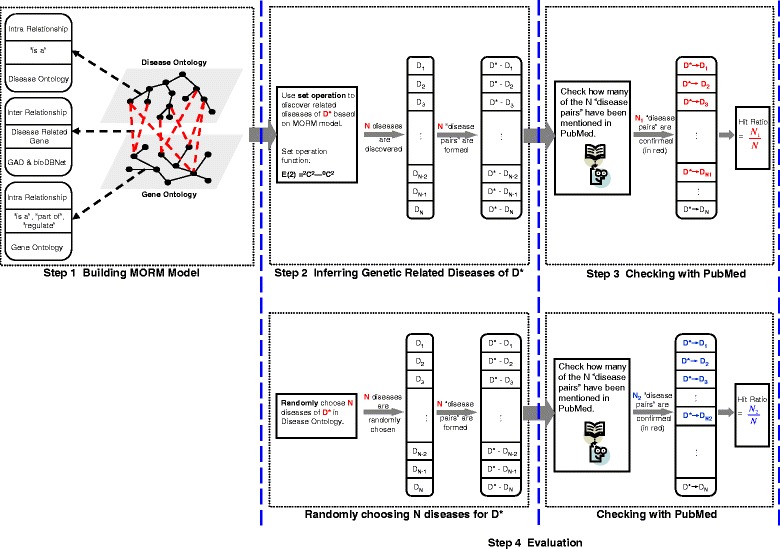
Flow chart of experimental design in experiment 2

**Step 1 MORM model building.** In the same way, a MORM model is built containing the same Disease Ontology (DO) and the Gene Ontology (GO), as shown in Fig. 4. GO is an ontology of defined terms representing gene product properties, which covers three domains: cellular components, molecular function and biological processes. There are three kinds of intra-relationships (“is-a”, “part of” and “regulate”), *R*_intra_, in GO. The two ontologies are linked through direct inter-relationships found using the Genetic Association Database (GAD), and the bioDBnet database. In Marwah’s work [10], the relationships between two ontologies are derived from the literature. In contrast, our approach is to build relationships from databases, and evaluate the experimental results by comparing with results reported in the literature.

Specifically, we first identify the mapping from disease terms to gene symbols in GAD, and then find the mapping from gene symbols to disease terms in bioDBnet. We finally create actual linkages (relationships) based on all the mapped pairs of disease terms and gene symbols between DO and GO. It should be pointed out that, in GO, some terms may connect to a great number of gene symbols, indicating such gene product properties may be needed for normal biological processes. When building inter-relationship between DO and GO, we exclude these kinds of gene product properties from our model.

The resulting linked ontology (linking DO and GO) contains 2214 inter-relationships between 800 genetic diseases in DO and 935 gene product properties in GO.

**Step 2 Inferring similar diseases** We now can apply our set-theoretic inference engine to build the relatedness network and explore the relatedness of one disease to other diseases within the network of relatedness at the gene level. Specifically, to discover related diseases that are not classified as similar diseases under current classification system, we compute the set *F*(2) for “anchor disease”, denoted as *D*^∗^, based on MORM model in step 1 (*F*(2)= ^2^*D*^2^− ^0^*D*^2^), and denote the number of discovered similar diseases as N. Then, we link *D*^∗^ with each of its similar diseases and form N “diseases pairs”, as shown in Fig. 4.

**Step 3 Checking with PubMed** To evaluate the effectiveness of the proposed model, we use already published cases and results in PubMed as references. As shown in Fig. 4, we compare the *N* “disease pairs” from step 2 with published cases in PubMed, and check how many of the “disease pairs” were confirmed by PubMed. This number is denoted as *N*1. Then, hit ratio is computed as *N*1/*N* (discovered “disease pairs” that confirmed in the PubMed, vs. the total discovered “disease pairs”).

**Step 4 Comparative evaluation with random method** To make a comparison, we implement a randomly pairing method. For each disease, we randomly choose *N* diseases and form *N* disease pairs accordingly, and see how many of these “disease pairs” are mentioned in the references. This number is denoted as *N*2. The hit ratio is *N*2/*N*. Then, we compare the two hit ratios for all the 800 genetics diseases.

#### Result analysis

**Fig. 5:**
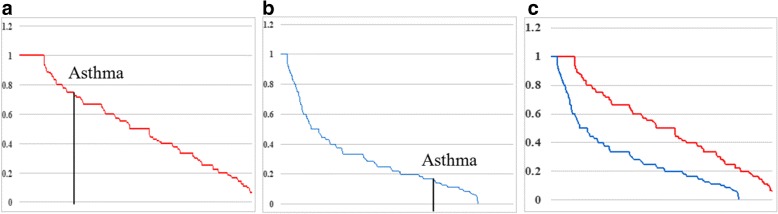
Hit ratio curves of the proposed method (**a**) and the randomly pairing method (**b**). The two curves are compared in (**c**)

The comparative evaluation of effectiveness of our method and the randomly pairing method is plotted in Fig. 5.

Figure 5a shows the hit ratio (*N*1/*N*) curve (in red) for all “disease pairs” discovered by our method, while Fig. 5b shows the hit ration (*N*1/*N*) curve (in blue) for all the “diseases pairs” discovered by randomly paring method, both in descending order. For example, we find 6 “disease pairs” of Asthma from the proposed model, 4 of which are confirmed in PubMed, giving a hit ratio of 0.67 (Fig. 5a), while the randomly pairing method only achieves a hit ratio of 0.17 for the same disease (Fig. 5b). Figure 5c presents a more intuitive comparison of the two methods by combining the two plots, which clearly shows the better performance of the proposed method.

### Experiment 3 discovering interesting relatednesses from BMKN using pruning strategy

We have recognized that within the multiple ontologies framework of MORM, a computed relatedness network can be very large often containing a great number of concepts and links, which still presents somewhat a challenge to review and disseminate the meaningful information from it. Considering the fact that, in problem-specific cases, not all relationships are interesting or useful for the tasks being pursued, we designed and implemented link (relationship) masks to prune the knowledge networks. In this experiment, a set of different link masks are applied to the original BMKN, and RN is computed afterwards. The effectiveness of pruning is evaluated.

#### Experimental design

**Fig. 6:**
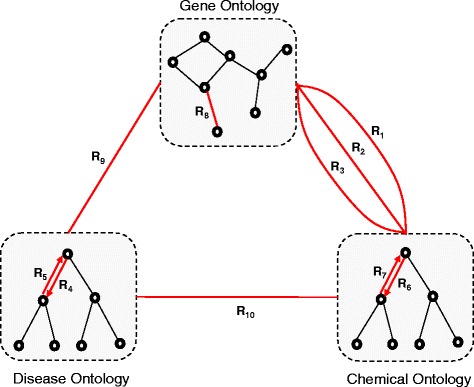
Biomedical knowledge network in experiment 3

We construct a BMKN, as shown in Fig. 6, from several knowledge repositories (DO, GO, CTD, DrugBank, KEGG, GenBank, STRING). Based on BMKN, we build a MORM containing concepts of diseases, genes and drugs (chemicals), as well as inter- and intra-relationships between them. There are total 10 relationships both within and across ontology systems as shown in Table 2.

**Table 2:** Semantic relationships of BMKN

R1: “affects” of gene - chemical
R2: “decrease” of gene - chemical
R3: “increase” of gene - chemical
R4: “father to son” of disease ontology
R5: “son to father” of disease ontology
R6: “father to son” of chemical ontology
R7: “son to father” of chemical ontology
R8: “interact” of gene ontology
R9: “affect” of gene - disease
R10: “affect” of disease - chemical

To help find potentially interesting chemicals for a particular disease, link masks are first applied to the original ontology network, and a relatedness network is then computed for the pruned knowledge network.

For the first part of the experiment, assuming we are only interested in indirect and implicit relatedness, the direct relationship between diseases and chemicals (R10) are to be pruned, represented as $F(i) = {O_{t}^{i}} - {~}^{0}{O_{t}^{i}}$. We also set the number of expansion steps to 2, i.e. *i*=2. “Interesting” chemicals discovered for a specific disease is represented as *F*(2), after pruning direct links between diseases and chemicals. The result is shown in the first line of Table 3.

**Table 3:** Result of pruning strategy

	Pruning mask	Edges	Average count	Average percentage
No Mask	*R*_*all*_(1,0,0)^a^	971,585	1054.6	100 %
Mask 1	*R*_1_(0,0,0),*R*_*rest*_(1,0,0)^b^	969,001	566	26.78 %
Mask 2	*R*_2_(0,0,0),*R*_*rest*_(1,0,0)	971,501	460.5	22.77 %
Mask 3	*R*_3_(0,0,0),*R*_*rest*_(1,0,0)	964,792	90.7	4.22 %
Mask 4	*R*_4_(0,0,0),*R*_*rest*_(1,0,0)	965,000	124	46.65 %
Mask 5	*R*_5_(0,0,0),*R*_*rest*_(1,0,0)	965,000	66.6	8.94 %
Mask 6	*R*_6_(0,0,0),*R*_*rest*_(1,0,0)	810,489	1.5	0.21 %
Mask 7	*R*_7_(0,0,0),*R*_*rest*_(1,0,0)	810,489	954.6	53.17 %
Mask 8	*R*_9_(0,0,0),*R*_*rest*_(1,0,0)	965,189	27.9	2.96 %
Mask 9	*R*_*all*_(1,0,0.4)	965,718	753.3	63.01 %
Mask 10	*R*_8_(1,0.4,0)	382,972	4.5	0.24 %
Mask 11	*R*_1_(0,0,0),*R*_*rest*_(1,0.2,0.35)	615,921	136.7	19.56 %
Mask 12	*R*_5_(0,0,0),*R*_*rest*_(1,0.2,0)	615,179	476.5	20.57 %

^a^ *R*_*all*_ indicates all types of relationships

^b^ *R*_*rest*_ indicates rest of the types of relationships

After the first step of pruning, the remaining knowledge network may still be very large. To further constrain the search space, we use pruning mask strategy described in section 4.2. As shown in Table 3, we define 12 pruning masks. Each mask, as shown in column 2, is a triplet representing semantic relationships value, confidence value and significance value. Each link mask is applied to the knowledge network, and the number of links in the pruned knowledge network is given in Column 3. Finally, we apply MORM on sub-networks for each disease concept as the anchor concept, and calculate the average counts of discovered chemicals for all disease concepts in DO, which are shown in column 4. Then, we compare the average counts based on the pruned network and average counts based on non-masking knowledge network, and calculate the average percentage.

#### Result analysis

From the results in Table 3 we can see that pruning masks do a great job on constraining search space. By using pruning masks, both counts and percentages of interesting chemical candidates are greatly decreased, indicating the effectiveness of pruning masks. It should be pointed out that, pruning masks should be set and designed by users for their own specific tasks or purposes. In this experiment, we set values of pruning masks on an acceptable level to extract relatively important links. However, when users choose their own values of pruning masks, results may vary under different situations, thus needs further research for specific field.

## Conclusion

In this paper, a novel unified computational framework is proposed in terms of a network of linked biomedical entities across multiple knowledge and information sources, consisting of biomedical ontologies and other biomedical repositories such as PubMed. This biomedical knowledge network (BMKN) is constructed using inter-relationships between concepts from different and information sources (e.g. ontologies, publication repositories). Within BMKN, a Multi-Ontology Relatedness Model (MORM) is developed, which includes the formation of multiple related ontologies, a relatedness network and a formal inference mechanism based on set-theoretic operations. Based on MORM, the inference engine computes interesting relatedness between concepts appeared in ontologies for which potential valuable relationships (implicit knowledge) may exist. Various problem-specific link masks can be designed to prune the original knowledge networks and computed relatedness networks to reduce the search space. Experimental results with example applications demonstrate the promising potential of our approach for discovering implicit knowledge in biomedical knowledge networks. However, we want to emphasize that the relatedness network computed by our method are a set of potentially valuable biomedical concepts and their relationships. They are the candidates or targets for deeper investigation in the context of specific biomedical domains. We are currently developing a set of relatedness network-guided data mining and knowledge discovery algorithms within the same framework. For example, some concepts in a relatedness network possess an interesting property of high degree of connectivity (i.e. have direct relationships with many other concepts) like “hub” nodes. We propose to study whether this type of concepts play a special role in implicit knowledge discovery.
